# A Retrospective Exploratory Analysis for Serum Extracellular Vesicles Reveals APRIL (TNFSF13), CXCL13, and VEGF-A as Prognostic Biomarkers for Neoadjuvant Chemotherapy in Triple-Negative Breast Cancer

**DOI:** 10.3390/ijms242115576

**Published:** 2023-10-25

**Authors:** Hae Hyun Jung, Ji-Yeon Kim, Eun Yoon Cho, Jeong Eon Lee, Seok Won Kim, Seok Jin Nam, Yeon Hee Park, Jin Seok Ahn, Young-Hyuck Im

**Affiliations:** 1 Department of Health Sciences and Technology, Samsung Advanced Institute for Health Sciences and Technology, Sungkyunkwan University, Seoul 06351, Republic of Korea; junghh0616@gmail.com (H.H.J.); jyeon25.kim@samsung.com (J.-Y.K.); yhparkhmo@skku.edu (Y.H.P.); 2 Biomedical Research Institute, Samsung Medical Center, Seoul 06351, Republic of Korea; 3 Division of Hematology-Oncology, Department of Medicine, Samsung Medical Center, Seoul 06351, Republic of Korea; ajis@skku.edu; 4 School of Medicine, Sungkyunkwan University, Suwon 16419, Republic of Korea; eunyoon.cho@samsung.com (E.Y.C.); paojlus@hanmail.net (J.E.L.); seokwon1.kim@samsung.com (S.W.K.); seokjin.nam@samsung.com (S.J.N.); 5 Department of Pathology, Samsung Medical Center, Seoul 06351, Republic of Korea; 6 Department of Surgery, Samsung Medical Center, Seoul 06351, Republic of Korea

**Keywords:** extracellular vesicles, exosomes, triple-negative breast cancer, neoadjuvant chemotherapy, prognostic biomarker, residual cancer burden, A proliferation-inducing ligand, C-X-C motif chemokine ligand 13, vascular endothelial growth factor A

## Abstract

Neoadjuvant chemotherapy (NAC) is widely used as a standard treatment for early-stage triple-negative breast cancer (TNBC). While patients who achieve pathologic complete response (pCR) have a highly favorable outcome, patients who do not achieve pCR have variable prognoses. It is important to identify patients who are most likely to have poor survival outcomes to identify candidates for more aggressive therapeutic approaches after NAC. Many studies have demonstrated that cytokines and growth factors packaged into extracellular vesicles (EVs) have an essential role in tumor progression and drug resistance. In this study, we examined the role of serum-derived EV-associated cytokines as prognostic biomarkers for long-term outcomes in patients who underwent anthracycline–taxane-based NAC. We isolated extracellular vesicles from the serum of 190 TNBC patients who underwent NAC between 2015 and 2018 at Samsung Medical Center. EV-associated cytokine concentrations were measured with ProcartaPlex Immune Monitoring 65-plex panels. The prognostic value of EV-associated cytokines was studied. We found that patients with high EV_APRIL, EV_CXCL13, and EV_VEGF-A levels had shorter overall survival (OS). We further evaluated the role of these selected biomarkers as prognostic factors in patients with residual disease (RD) after NAC. Even in patients with RD, high levels of EV_APRIL, EV_CXCL13, and EV_VEGF-A were correlated with poor OS. In all subgroup analyses, EV_CXCL13 overexpression was significantly associated with poor overall survival. Moreover, multivariate analysis indicated that a high level of EV_CXCL13 was an independent predictor of poor OS. Correlation analysis between biomarker levels in EVs and serum showed that EV_VEGF-A positively correlated with soluble VEGF-A but not CXCL13. An elevated level of soluble VEGF-A was also associated with poor OS. These findings suggest that EV_APRIL, EV_CXCL13, and EV_VEGF-A may be useful in identifying TNBC patients at risk of poor survival outcomes after NAC.

## 1. Introduction

Triple-negative breast cancer (TNBC), which is defined by the lack of estrogen receptor (ER), progesterone receptor (PR), and human epidermal growth factor receptor 2 (HER2) expression, accounts for 15–20% of all breast cancers. TNBC is associated with a younger age of onset, more advanced stage, and poor prognosis compared to non-TNBCs. Due to the lack of therapeutic targets, cytotoxic chemotherapy is the only recommended systemic treatment strategy for patients with TNBC [1,2].

Neoadjuvant chemotherapy (NAC) is widely used as a standard treatment option for early, operable TNBC patients. Pathologic complete response (pCR) after NAC is a powerful predictor of favorable long-term outcomes [3,4]. However, patients with residual disease (RD) after NAC have heterogeneous diseases with diverse prognoses, which lead to a higher risk of relapse and poor prognosis compared to those with pCR [5,6,7]. The Residual Cancer Burden (RCB) index was developed to quantify residual disease following NAC and has been validated as a predictor of long-term survival. In patients with TNBC, those with pCR and RCB I have a good prognosis, while those with RCB II and III show a decreased likelihood of survival [8,9]. It is important to sub-classify patients into different prognostic groups to find candidates for more aggressive therapeutic approaches after NAC. Several studies have suggested that the combination of the RCB index and additional factors such as lymphovascular invasion [10], tumor-infiltrating lymphocytes [11,12], PD-L1 expression [13], or Ki67 expression after NAC [14,15] could improve the prognostic prediction. However, these methods are tumor tissue-based approaches that do not provide comprehensive information about the status of cancer, which makes it difficult to readily introduce them into the clinic.

Liquid biopsy-based biomarkers, including circulating proteins, circulating tumor cells (CTCs), circulating tumor DNA (ctDNA), and extracellular vesicles (EVs), have recently been suggested as minimally invasive biomarkers for diagnostics, prognosis prediction, and therapy response monitoring [16]. However, the clinical utility of CTCs and ctDNA is limited because of their short life span and low concentration. EVs are nano-sized membranous structures that are released by various cells into biological fluids such as plasma and urine. EVs contain various bioactive molecules (nucleic acids, protein, lipid) and are enriched with specific cancer-associated contents. Therefore, many studies investigating liquid biopsies have focused on EVs [17].

Several studies have reported that EV contents have the potential to be biomarkers for the diagnosis and prognosis of TNBC. One study demonstrated that the serum level of EV-miR-373 was significantly upregulated in patients with TNBC compared to that in patients with non-TNBC [18]. Another study reported that the expression level of EV-lncRNA small ubiquitin-like pseudogene 3 (SUMO1P3) was significantly higher in patients with TNBC than in those with non-TNBC and healthy controls; its upregulation was significantly correlated with poor survival [19]. Lan et al. found that serum EV-LncRNA X-Inactive Specific Transcript (XIST) decreased significantly after primary breast tumor resection and increased at recurrence of TNBC [20]. Also, serum EV-annexin A2 was shown to have prognostic value with a positive correlation to the tumor grade of TNBC and poor overall survival [21]. In addition, some reports suggest that circulating EVs have predictive value for assessing NAC responses in breast cancer [22,23,24,25]. In a randomized phase II neoadjuvant GeparSixto trial, Stevic et al. showed that some miRNAs were selectively enriched in the EVs of HER2+ BC and TNBC and that EV-miR-155 and EV-miR-301 were good candidates for predicting pCR [26]. Another study showed that breast cancer resistance protein (BCRP) was elevated in plasma EVs isolated from NAC-resistant BC patients [27]. However, few studies have elucidated the prognostic value of circulating EVs in breast cancer patients undergoing NAC.

Circulating proteins such as cytokines and growth factors play an important role in the development and progression of breast cancers and are promising biomarkers of chemotherapy response. Several studies have indicated that interleukin-6 (IL-6) and interleukin-8 (IL-8) secretion promote multiple drug resistance while blocking these cytokines inhibits tumor cell viability and migration [28,29]. One study evaluated the prognostic and predictive values of serum transforming growth factor-β (TGF-β) and vascular endothelial growth factor A (VEGF-A) in TNBC [30]. Recently, Fitzgerald et al. reported that cytokines could be released not only in soluble but also in EV-associated form [31]. One study showed that EV-associated VEGF stimulated tumor growth but was not neutralized by bevacizumab [32]. These data indicate that EV-associated cytokines are an attractive potential source of biomarkers that could predict the long-term outcomes of patients who receive NAC.

Previously, we reported the clinical relevance of EV-associated cytokines in breast cancer. We found that elevated nerve growth factor (NGF) in serum EV was related to poor survival outcomes in BC patients treated with NAC. The previous study was conducted in all subtypes of BC, mainly in hormone receptor-positive breast cancer [33]. In the present study, we evaluated the expression of EV-associated cytokines focusing on TNBC patients who received NAC. We investigated the correlations of these cytokines with clinical characteristics and patient prognosis. We further assessed the prognostic value of EV-associated cytokines according to the RCB class or TNM stage.

## 2. Results

### 2.1. Patient Characteristics

A total of 190 patients with TNBC who received neoadjuvant AC-T (anthracycline + cyclophosphamide, followed by taxane) chemotherapy were included in this study (Figure S1). The patient characteristics and clinical outcomes are summarized in Table 1. The mean age at diagnosis was 49 years (range 23–70 years), and 19.5% (*n* = 37) in patients younger than 40 years. One hundred patients (52.7%) were premenopausal. Ninety-four patients (49.5%) had preoperative clinical stage II disease, while 96 patients (50.6%) had clinical stage III disease. One hundred thirty-four patients (70.6%) were classified as clinical T2 and 37 patients (19.5%) as clinical T3. Clinically, axillary lymph node involvement was present in 150 patients (78.9%).

The overall pCR rate after NAC was 34.3% (65 patients). Sixty-one patients (32.2%) were classified as postoperative pathologic stage I, 44 patients (23.3%) as pathologic stage II, and 20 patients (10.6%) as pathologic stage III. One hundred and sixteen (61.1%) patients presented with nuclear grade 3, and 82 (43.2%) patients presented with histologic grade 3. The RCB scores or classes were assessed in 117 of 125 tumors. The proportion of patients within each RCB class was as follows: RCB-I, *n* = 21 (11.1%); RCB-II, *n* = 71 (37.4%); and RCB-III, *n* = 25 (13.2%).

Within a median follow-up of 5 years (range from 0.3 to 6.7), 45 patients (23.7%) had relapsed, including 43 patients (22.6%) with distant relapse and 12 patients with locoregional relapse. Twenty-five patients (13.2%) died during the follow-up period. The survival outcomes according to patient characteristics are reported in Table 1.

### 2.2. Characterization of EVs Isolated from Serum

**Figure 1:**
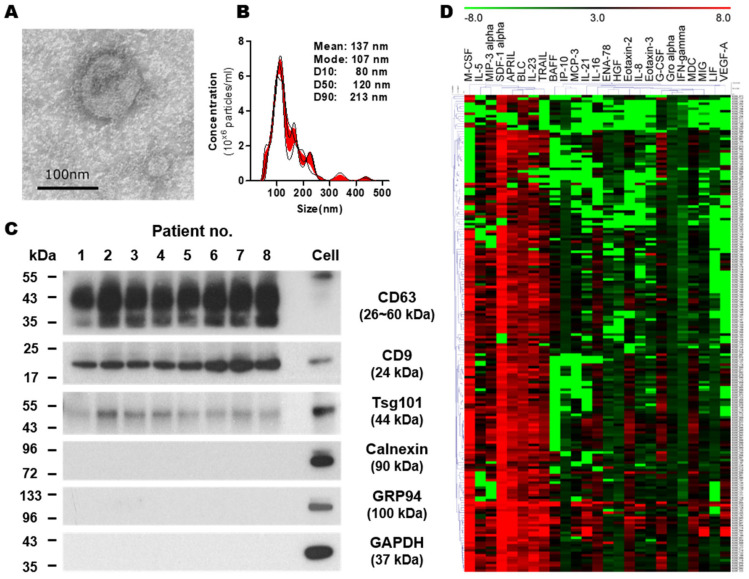
Characterization of EVs in BC patients. (**A**) Representative TEM image of isolated EVs. Scale bar: 100 nm. (**B**) Nanoparticle tracking analysis. The calculated size distribution is depicted as mean (black line) with standard error (red shading). (**C**) Western blot analysis indicating the presence of EV markers (CD63, CD9, and Tsg101) and the absence of negative markers (Calnexin, GM130, and GAPDH) in the purified EVs. The lysate of an MCF7 cell line was loaded as a control. The uncropped blots of (**C**) are shown in Figure S2. (**D**) Heatmaps of detected proteins in EV lysates. Heat map demonstrating unsupervised hierarchical clustering of samples generated with a Multi-Experiment Viewer (MeV v4.9).

We isolated EVs from the patient’s serum using a sequential ultracentrifugation method. To verify the EVs isolated from the serum of BC patients, we validated them in terms of morphology, size, and specific markers. TEM analysis demonstrated spherical, membrane-bound vesicles (Figure 1A). The particle size measurement via NTA analysis validated the average size of the EV population at 106.8 ± 5.9 nm (Figure 1B). Furthermore, these EVs were positive for the typical EV markers CD63, CD9, and TSG101, while calnexin, GM130, and GAPDH were absent (Figure 1C). Altogether, these results showed that our EVs were successfully generated from the serum samples of BC patients.

Next, we measured the levels of 65 analytes in lysed EVs using the ProcartaPlex Human Immune Monitoring 65-Plex Panel (Thermo Fisher Scientific, Waltham, WA, USA). The expression profiles of biomarkers in EVs are described in Figure 1D and Table S1. The data showed that 35 analytes were detected in EVs. The following cytokines in serum EVs had relatively higher levels (mean > 50 pg/mL) than other analytes: M-CSF; SDF-1α; APRIL; IL-23; TRAIL; and CXCL13. Thirty analytes (CD40L, FGF-2, fractalkine, GM-CSF, IL-1α, IL-1β, IL-10, IL-12p70, IL-13, IL-15, IL-17A, IL-2, IL-22, IL-27, IL-2R, IL-3, IL-31, IL-4, IL-6, IL-7, IL-9, I-TAC, MCP-1, MCP-2, MIP-1α, MIP-1β, MMP-1, NGF-β, TNF-β, and TSLP) were not included in this study since the majority (>80% samples) of results were below the limit of assay sensitivity.

### 2.3. Prognostic Value of EV Biomarkers 

In order to investigate the prognostic values of the EV analytes for BC survival, we performed receiver operating characteristic (ROC) curve analysis. Among the 35 biomarkers tested, only three (APRIL, CXCL13, and VEGF-A) were significantly correlated with OS (Table 2). The ROC characteristics of the three significant biomarkers were EV_APRIL (AUC = 0.630, *p* = 0.036), EV_CXCL13 (AUC = 0.671, *p* = 0.006), and EV_VEGF-A (AUC = 0.698, *p* = 0.001). The optimal cutoff values were calculated using the Youden index method. Furthermore, comparative analysis of biomarker expression according to survival demonstrated significantly higher cytokine levels in the dead group than in the live group (Figure S3).

**Figure 2:**
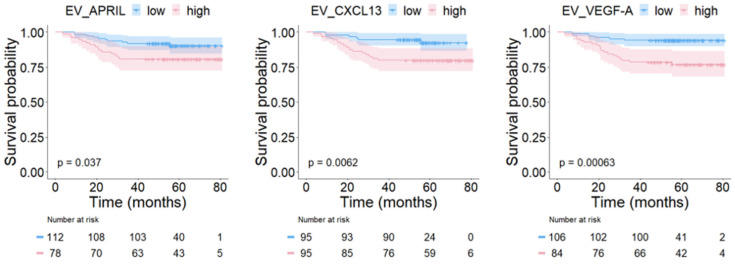
Kaplan–Meier curves for overall survival according to the level of biomarker expression. High levels of EV_APRIL, EV_CXCL13, and EV_VEGF-A were significantly associated with poor OS.

As shown in Figure 2, patients with high expression of EV_APRIL (80.8% vs. 91.1%; *p* = 0.037), EV_CXCL13 (80.0% vs. 93.7%; *p* = 0.006), and EV_VEGF-A (77.4% vs. 94.3%; *p* < 0.001) had shorter OS than the low expression group.

### 2.4. Relationships between the Level of Potential Biomarkers and the Clinicopathological Parameters of Patients with Breast Cancer

We further evaluated the relationships between the selected biomarkers and clinical characteristics (including age, menopausal status, clinical stage, TNM stage, and RCB class). We did not find a significant association between the biomarkers and patient age or menopausal status. A high EV_APRIL level was positively correlated with unfavorable parameters such as the postoperative pathologic stage (*p* = 0.01). Likewise, overexpression of EV_CXCL13 also was positively correlated with high RCB Class (*p* = 0.012). Elevated EV_VEGF-A expression significantly correlated with the clinical stage (*p* = 0.012) and clinical node status (*p* = 0.004) but not with RCB class (*p* = 0.067). These results are summarized in Table 3.

**Figure 3:**
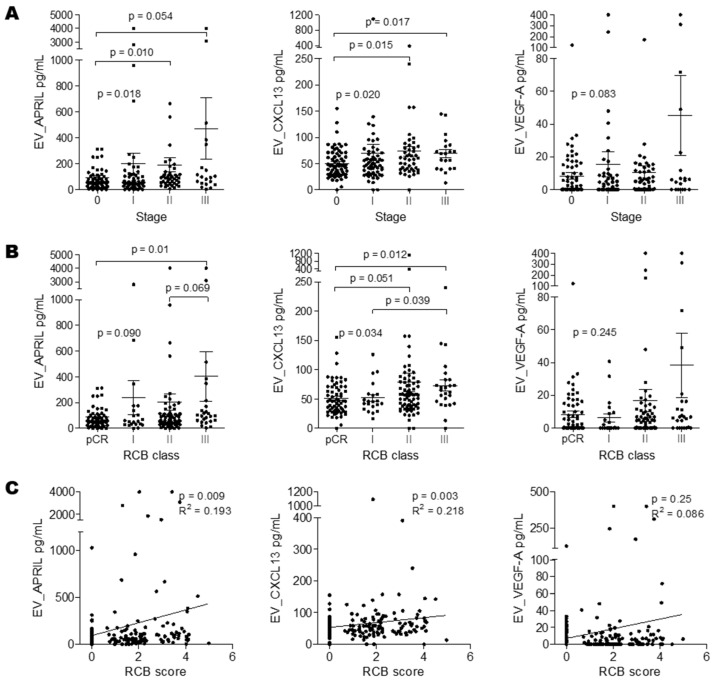
Biomarker expression levels according to stage and RCB. Scatter plot showing the distribution of biomarker levels according to stage (**A**) and RCB class (**B**). The differences between groups were evaluated with the Kruskal–Wallis test, and pairwise comparisons were performed with the Mann–Whitney *U* test. (**C**) Correlation plots of the RCB score and biomarker level. Spearman’s correlation coefficient was used for correlation analysis.

The comparative analysis of biomarker expression according to TNM stage demonstrated significantly higher levels of EV_APRIL (*p* = 0.018) and EV_CXCL13 (*p* = 0.020) in patients with stages II and III compared to those of patients with pCR (Figure 3A). When we analyzed the biomarker expressions in patients stratified according to RCB class, EV_ CXCL13 was overexpressed in RCB III vs. pCR (*p* = 0.012) and in RCB III vs. RCB I (*p* = 0.039) (Figure 3B). We further analyzed the relationships between the RCB score and the expression levels of biomarkers via Spearman’s rank correlation analysis. As shown in Figure 3C, the RCB score was weakly associated with EV_APRIL (r = 0.193; *p* = 0.009) and EV_CXCL13 level (r = 0.218; *p* = 0.003). 

### 2.5. Prognostic Value of EV Biomarkers in Patients with Non-pCR, RCB II/III, and Stages II/III 

**Figure 4:**
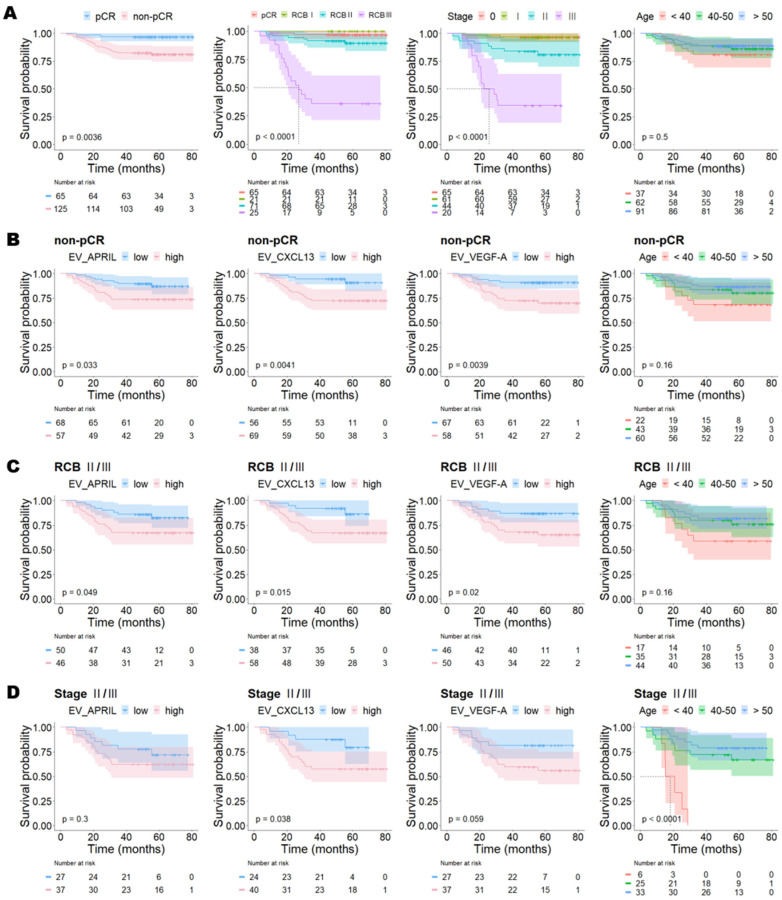
Kaplan–Meier plots for overall survival in patients with different stages and RCB classes. Kaplan–Meier plots for overall survival in all patients (**A**), patients with non-pCR (**B**), patients with RCB II/III tumors (**C**), and patients with stage II/III tumors (**D**).

Of the 190 included patients, 25 (13.2%) died during follow-up. The 5-year overall survival according to RCB class and stage is shown in Table 1 and Figure 4A. The 5-year OS rates of the groups with RCB 0, RCB I, RCB II, and RCB III were 96.9%, 100%, 90.1%, and 36.1%, respectively. The 5-year OS rates in patients with stage 0, IA, and IB disease were 96.9%, 96.4%, and 100%, respectively. In contrast, the 5-year OS rates in patients with stage IIA, IIB, IIIA, and IIIC disease were 93.5%, 53.8%, 38.5%, and 28.6%, respectively. 

Because RCB class and disease stage significantly affected OS, we further stratified BC patients by both RCB class (pCR vs. non-pCR or RCB 0/I vs. RCB II/III) and stage (stage 0/I vs. stage II/III). The 5-year OS rate was lower in non-pCR than it was in pCR (81.6% vs. 96.9%; *p* = 0.004); RCB II/III compared with RCB 0/I (76.0% vs. 97.7%; *p* < 0.001); and stages II/III versus stages 0/I (67.2% vs. 96.8%; *p* < 0.001) (Figure 4A). 

Next, we analyzed the relationships between the three biomarker levels and OS according to divided subgroups. In patients with pCR, RCB 0/I, and stage 0/I, the biomarker expression levels did not affect patient survival. 

However, in the non-pCR group, patients with overexpression of EV_APRIL (73.7% vs. 88.2%; *p* = 0.033), EV_CXCL13 (72.5% vs. 92.9%; *p* = 0.004), and EV_VEGF-A (70.7% vs. 91.0%; *p* = 0.004) had significantly poorer survival outcomes than those with low expression (Figure 4B). In RCB II/III breast cancer, patients with high expression of EV_APRIL (67.4% vs. 84.0%; *p* = 0.049), EV_CXCL13 (67.2% vs. 89.5%; *p* = 0.015), and EV_VEGF-A (66.0% vs. 87.0%; *p* = 0.020) had shorter OS than those with low cytokine expressions (Figure 4C). In the advanced-stage group (stages II/III), patients with elevated EV_CXCL13 expression (57.5% vs. 83.3%; *p* = 0.038) had significantly poorer survival outcomes than those with low EV_CXCL13 expression. The level of EV_VEGF-A expression also marginally impacted the OS (56.8% vs. 81.5%, *p* = 0.059) (Figure 4D). 

We analyzed the relationship between age and OS in the high RCB and advanced-stage groups. In RCB II/III breast cancer, the 5-year OS rates in patients aged <40, 40–50, and >50 years were 58.8%, 77.1%, and 81.8%, respectively. Younger patients tended to have lower survival rates than older patients, although this was not significant. In the advanced-stage group (stages II/III), patients aged <40 years had the worst survival rate of 0% compared to 68.0% and 78.8% for the 40–50 and >50 groups, respectively (*p* < 0.0001) (Figure 4).

We then performed a Cox proportional hazard analysis to evaluate the prognostic value of EV_APRIL, EV_CXCL13, and EV_VEGF-A, along with key clinicopathological features (Table 4). In the non-pCR group, univariate analysis showed that the TNM stage, EV_APRIL level, EV_CXCL13 level, and EV_VEGF-A level were significantly associated with OS (*p* < 0.001, *p* = 0.039, *p* = 0.008, and *p* = 0.007, respectively). Multivariate analysis revealed that age (<40 vs. >50, HR = 4.76, *p* = 0.008), TNM stage (*p* < 0.001), and EV_CXCL13 level (HR = 4.33, *p* = 0.011) were independent predictors of poor OS. In RCB II/III breast cancer, univariate analysis showed that the TNM stage, EV_CXCL13 level, and EV_VEGF-A level were correlated with OS (*p* < 0.001, *p* = 0.023, and *p* = 0.026, respectively). Multivariate analysis indicated that age (<40 vs. >50, HR = 4.57, *p* = 0.010), TNM stage (*p* < 0.001), and EV_CXCL13 level (HR = 4.17, *p* = 0.014) had prognostic effects on the survival outcomes. In the advanced-stage group (stages II/III), univariate analysis showed that age, TNM stage, and EV_CXCL13 level were significant predictors for OS (*p* = 0.001, *p* < 0.001, and *p* = 0.048, respectively). Moreover, a multivariate analysis demonstrated that age (<40 vs. >50, HR = 5.59, *p* = 0.006), TNM stage (*p* = 0.001), and EV_CXCL13 level (HR = 4.05, *p* = 0.019) were independent predictors of poor OS.

In all three groups, age, stage, and EV_CXCL13 were independent prognostic factors for poor OS in the Cox model.

### 2.6. Correlation Analysis among Biomarkers Detected in EVs and Serum

**Figure 5:**
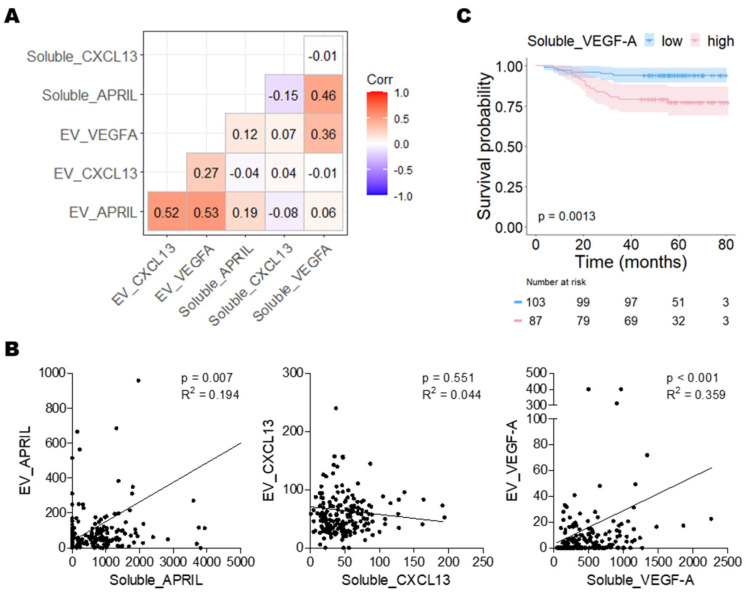
Correlation analysis among biomarkers detected in the EVs and serum. (**A**) Correlation analysis among APRIL, CXCL13, and VEGF-A in the EVs and serum. The color scale represents the correlation, and *p*-values are shown in Table S2. (**B**) Correlation plots between biomarker levels in the EVs and serum. Spearman’s correlation coefficient was used for correlation analysis. (**C**) Kaplan–Meier curves for overall survival. High expression of soluble VEGF-A was significantly associated with poor OS.

To confirm the difference in biomarker expression in EVs and serum, we further analyzed the serum concentrations of soluble APRIL, CXCL13, and VEGF-A using a multiplex immunoassay. The average concentrations of the three biomarkers in the serum of BC patients were 1714.3 ± 6271.4 pg/mL of soluble APRIL, 49.4 ± 32.9 pg/mL of soluble CXCL13, and 464.7 ± 359.1 pg/mL of soluble VEGF-A. Interactions among the biomarkers detected in the EVs and serum were analyzed via Spearman correlation analysis (Figure 5A, Table S2). With regard to EV-associated biomarkers, APRIL expression was positively correlated with CXCL13 and VEGF-A expression (Spearman R between APRIL and CXCL13: 0.520, *p* < 0.001, and Spearman R between APRIL and VEGF-A: 0.531, *p* < 0.001), and CXCL13 was positively correlated with VEGF-A expression (Spearman R between CXCL13 and VEGF-A: 0.271, *p* < 0.001). With regard to soluble biomarkers, APRIL expression was negatively correlated with CXCL13 (Spearman R between APRIL and CXCL13: -0.149, *p* = 0.040) but positively correlated with VEGF-A expression (Spearman R between APRIL and VEGF-A: 0.462, *p* < 0.001). Moreover, a significant correlation was found between the relative expression of APRIL and VEGF-A in the EV and serum (Spearman R between EV_APRIL and soluble_APRIL: 0.194, *p* = 0.007, and Spearman R between EV_VEGF-A and soluble_VEGF-A: 0.359, *p* < 0.001) but not for CXCL13 (*p* = 0.551) (Figure 5B).

Subsequently, a receiver operating characteristic (ROC) curve was constructed to assess the prognostic value of the soluble protein in the patients’ serums. Among the three soluble biomarkers, only VEGF-A had statistical significance (AUC = 0.684, *p* = 0.003, cutoff = 381.50) (Figure S4). Based on the obtained cutoff values, patients with high levels of soluble VEGF-A had poorer OS than those with low levels (78.2% vs. 94.2%; *p* = 0.001) (Figure 5C).

## 3. Discussion

Neoadjuvant chemotherapy has proven useful at reducing the tumor burden preoperatively to facilitate breast conservation, rendering locally advanced cancers operable and eradicating micrometastases. However, the impact of NAC on prognosis is highly dependent on the achievement of pCR, especially in the TNBC [4]. The conventional NAC regimen composed of adriamycin, cyclophosphamide, and taxane (paclitaxel or docetaxel) (AC-T) yields pCR rates of 35–45% [34,35]. While patients who achieve pCR have a highly favorable outcome, patients who do not achieve pCR have very diverse prognoses [7]. Reliable biomarkers for predicting the long-term clinical outcome of patients after NAC are not yet well-established. Therefore, there is a need to identify new biomarkers for this purpose.

In this study, we examined the role of serum-derived EV-associated cytokines as prognostic biomarkers for the long-term outcomes of patients who underwent NAC. We found that patients with high levels of EV_APRIL, EV_CXCL13, and EV_VEGF-A had shorter overall survival than those with low levels of these cytokines.

The RCB class is a pathologic tumor staging system for characterizing residual tumors when patients fail to achieve pCR after NAC. Some studies have shown that patients achieving RCB-I have a similar prognosis to those achieving pCR, whereas patients with RCB-II and RCB-III have a poor prognosis [8,9]. We similarly found that patients who achieved pCR (5Y-OS: 96.9%) or RCB-I (5Y-OS: 100%) had good prognoses in our cohort. Campbell et al. compared the recurrence outcomes based on RCB class and TNM stage in patients enrolled in the I-SPY 1 trial. The authors reported discrepancies between RCB and TNM stratification and that there was a benefit in reporting both the RCB class and TNM stage for patients in order to identify those at the highest risk of recurrence [36]. One study evaluated survival and distant recurrence by BC subtype in patients with residual stages II/III after NAC. They reported that patients with TNBC had a significantly poorer survival outcome than patients with other breast cancer types [37]. Ren et al. reported that high CD3+ and CD4+ signals in the tumor area were significantly associated with better survival, even in advanced stages (stages IIB/III) TNBC [38].

Based on these studies, we further evaluated the role of EV-associated cytokines as prognostic values in patients with residual disease (non-pCR), RCB II/III, and stages II/III. In 190 patients enrolled, the proportions of patients with non-pCR, RCB II/III, and stages II/III were 65.8% (*n* = 125), 50.5% (*n* = 96), and 33.7% (*n* = 64), respectively. In the non-pCR group, we also demonstrated that patients with high levels of EV_APRIL, EV_CXCL13, and EV_VEGF-A had shorter overall survival than those with low levels of these cytokines. In all subgroup analyses, we found that EV_CXCL13 overexpression was significantly associated with poor overall survival. Moreover, multivariate analysis indicated that young age, high stage, and high level of EV_CXCL13 were independent predictors of poor OS.

Young age at diagnosis is associated with poor prognosis in breast cancer patients. Previous studies reported that patients < 40 years of age have a poor histologic grade and a higher proportion of TNBC than older patients [39,40,41]. The prognostic impact of age on TNBC is controversial. Some studies found that young TNBC patients have an aggressive disease course [42,43]. In contrast, other papers reported that the prognosis of young patients did not differ from that of older patients in TNBC [44,45]. In the present study, the 5-year OS rates in patients aged <40, 40–50, and >50 years were 81.1%, 87.1%, and 89.0%, respectively (*p* = 0.498). However, we found that young age was correlated with poor survival in patients with non-pCR, RCB II/III, and stages II/III in multivariate analysis. The young age group (<40 years) in this study was identified in 37 women, of whom 7 died. All seven patients who died had RCB II/III tumors, while six patients had advanced stage (stages II/III) tumors. These results suggest that young age should be considered an unfavorable prognostic factor in TNBC patients with RCB II/III and advanced stage after NAC.

A proliferation-inducing ligand (APRIL), also known as tumor necrosis factor ligand superfamily member 13 (TNFSF13), is a member of the TNF ligand superfamily. APRIL plays a critical role in maintaining B cells and humoral immunity in the immune system but is also expressed in various cancers and substantially impacts tumor cell development and metastasis in hematological and solid cancers [46]. In particular, Garcia-Castro et al. demonstrated that APRIL correlated with TNBC and induced breast tumor proliferation and metastasis [47]. In addition, silencing of the APRIL receptor (TNFRSF13B) in the TNBC cell line induced significant cell death, indicating the APRIL system as a potential therapeutic target for the TNBC [48]. APRIL serum level was higher in patients with brain tumors in comparison to healthy individuals [49]. In addition, increased EV_APRIL level was strongly correlated with short progression-free survival of patients with metastatic urothelial carcinoma [50].

The C-X-C motif chemokine ligand 13 (CXCL13), also called B lymphocyte chemoattractant (BLC), is expressed by stromal cells within B cell follicles. It plays a role in B lymphocyte migration and accumulation by acting on its receptor, CXCR5 [51]. In recent years, several studies have reported the involvement of CXCL13 in the progression, metastasis, prognosis, apoptosis, and adaptive immunity of tumors, including breast cancer [52]. However, the roles of CXCL13 in breast cancer development and progression remain controversial. While some studies provided evidence that high CXCL13 expression was associated with an adverse prognosis [53,54], others showed opposite results [55,56]. Biswas et al. found that CXCL13-CXCR5 signaling induces the epithelial–mesenchymal transition of breast cancer cells and promotes lymph node metastasis [57]. A recent study demonstrated that CXCL13 contributes to tumor metastasis by affecting the recruitment of regulatory B cells [58]. Interestingly, Chen et al. reported that CXCL13 expression was higher in young Chinese BC patients (≤45 years) and was closely associated with lymph node positivity and ER-negative status [53]. There are rare studies that evaluate the value of CXCL13 as a blood-based biomarker in breast cancer. One study showed that serum CXCL13 level was overexpressed in patients with metastatic disease compared with those of healthy controls [59]. In contrast, other studies have failed to show elevated plasma CXCL13 levels in BC patients compared to those of normal controls despite differential CXCL13 expression in BC tissue and normal breast tissue [60]. As far as we know, our present article is the first study reporting that a high level of EV_CXCL13 is associated with poor survival in TNBC patients. However, we could not detect a significant correlation between the levels of soluble CXCL13 and EV_CXCL13. Soluble CXCL13 did not affect the survival outcome.

Vascular endothelial growth factor (VEGF, VEGF-A) is a major regulator of angiogenesis. VEGF signaling plays key roles in tumor growth, tumor angiogenesis, blood vessel permeability, and metastasis. VEGF is highly expressed in patients with TNBC compared with non-TNBC patients, and a high blood level of VEGF-A has been associated with a dismal prognosis [30,61]. Moreover, Wang et al. reported that serum VEGF is a biomarker that correlates with NAC response in TNBC, including the predictive value of pCR and disease-free survival. They found that a high level of VEGF was also associated with an unfavorable outcome [62]. Consistent with these studies, we observed an elevated level of soluble VEGF-A to be associated with poor prognosis. We also found that soluble VEGF-A has a strong correlation with EV_VEGF-A and that patients with high EV_VEGF-A had shorter overall survival compared to those with low levels.

Cytokine and growth factors are produced by a broad range of cells, including immune cells, endothelial cells, fibroblasts, and various stromal cells, and function via interacting with specific receptors on the target cell surface. APRIL is a cytokine produced primarily by myeloid cells, but cells of non-hematopoietic origin are also a potential source of APRIL [46]. APRIL is produced by malignant breast cancer cells [47]. Also, APRIL is released by neutrophils through the TLR4-PKR pathway activated by breast cancer [63]. CXCL13 is abundantly expressed on follicular helper T cells, follicular dendritic cells, and stromal cells in secondary lymphoid organs to guide CXCR5+ B and T cells from the blood into follicles. Within the tumor microenvironment, CXCL13 is secreted by multiple populations of cells, including lymphocytes, endothelial cells, stromal cells, and tumor cells [64]. A recent study showed that breast cancer cells expressing CCL21 induced CXCL13 secretion from stromal cells through the recruitment of innate lymphoid cells to the tumor microenvironment [65]. VEGF-A is most abundantly secreted by endothelial cells. In a state of hypoxia, VEGF-A is secreted by a variety of cells, including tumor cells, macrophages, platelets, dendritic cells, astrocytes, and osteoblasts [66]. VEGF-A is secreted into several isoforms with different biological properties through alternative splicing of mRNA, and the dominant isoform is VEGF165 [67]. In EVs studies, Ko and colleagues found that VEGF189 was preferentially enriched in small EVs that were secreted by cancer cells [32], and Feng and colleagues identified a unique 90 kDa form of VEGF on the surface of breast cancer cell-derived microvesicles [68]. These findings implicate that cytokines are packaged into EVs via different mechanisms, depending on the secreting cell and type of EV. For this reason, it is difficult to identify the primary source of EV-related cytokines in the blood. However, studies identifying the major sources of EV-associated cytokines and elucidating the mechanisms of action of the EV-associated cytokines in cancer progression are informative and warrant further experiments.

Cytokines and growth factors are present in both serum and EVs, and they can be released either in soluble or EV-associated form depending on the cell type, external stimuli, and physiological status [69]. Pathological conditions can also alter the amount and content of cytokines secreted into EVs. For instance, in human immunodeficiency virus-infected individuals or in diabetic patients, the profile of specific cytokines in EVs is significantly increased [70,71]. In this study, we found that CXCL13 and VEGF-A exhibit different expression patterns in EVs and serum, resulting in different survival outcomes. These results suggest that the formation of specific cytokines associated with EVs may affect breast cancer survival outcomes.

Our research is a retrospective study with a relatively small number of patients. However, the samples were homogeneous in terms of TNBC subtype and NAC regimen. In this study, we only measured cytokine expression at the time of curative surgery after NAC. Therefore, we could not provide information on whether baseline cytokine levels were associated with survival outcomes. 

In conclusion, we demonstrated that EV_APRIL, EV_CXCL13, and EV_VEGF-A may serve as predictors of OS in TNBC patients who undergo anthracycline–taxane-based NAC. Furthermore, the EV_CXCL13 level provides independent and additional prognostic information in TNBC patients with residual disease after NAC. Such information may help identify patients who will benefit from further adjuvant chemotherapy.

## 4. Materials and Methods

### 4.1. Patients

We retrospectively reviewed the medical data of patients diagnosed with clinical stages II to III BC who underwent NAC followed by curative surgery at the Samsung Medical Center between January 2015 and December 2018. Patients with bilateral BC, ductal carcinoma in situ, and distant metastases were excluded from this study. Of a total of 1295 patients, 824 patients had available serum samples at the Samsung Medical Center BioBank. Among 229 patients with the triple-negative phenotype, 190 TNBC patients who received neoadjuvant anthracycline/cyclophosphamide (AC) followed by taxane chemotherapy (AC-T) were included in this study. Of the 229 patients, 39 were excluded for homogeneous group analysis in terms of chemotherapy. Patients were excluded if they received the following regimens in NAC: anthracycline + cyclophosphamide (AC) (*n* = 10); anthracycline + taxane (AT, *n* = 1); or anthracycline + cyclophosphamide, followed by taxane + cisplatin or carboplatin (*n* = 15). Thirteen patients who received the following adjuvant chemotherapy regimens were also excluded: capecitabine (*n* = 11); docetaxel (*n* = 1); and cyclophosphamide plus methotrexate plus 5-fluorouracil (CMF, *n* = 1). The patient serum samples used in this project were provided by the Samsung Medical Center BioBank with informed consent from all donors. The process for selecting patients for biomarker development is presented in Supplementary Figure S1.

Pretreatment core biopsies and surgical specimens after surgery were reviewed by experienced pathologists. Pathologists determined the tumor histologic characteristics and receptor status (estrogen receptor [ER], progesterone receptor [PgR], and human epidermal growth factor receptor-2 [HER2]) according to hematoxylin and eosin (H&E) and immunohistochemical (IHC) staining. ER and PgR positivity were defined as an Allred score from 3 to 8 according to IHC staining with anti-ER (Immunotech, Marseille, France) and anti-PgR (Novocastra Laboratories Ltd., Newcastle upon Tyne, UK) antibodies, respectively. HER-2/neu status was evaluated using a specific antibody (Dako, Glostrop, Denmark), and grades 3 and 2 with the presence of amplification, confirmed via fluorescence in situ hybridization (FISH) or chromogenic in situ hybridization (CISH), were accepted as a positive result. TNBC was defined as a negative result for ER/PgR and ERBB2.

Pathologists determined the pathological response to NAC using surgical specimens. A pathological complete response (pCR) was defined as the loss of all invasive carcinoma cells in both breast and axillary lymph nodes (ypT0/Tis, N0) after completion of neoadjuvant chemotherapy [72]. The RCB score was assessed on surgical specimens of non-pCR patients using the Residual Cancer Burden (RCB) calculator on the MD Anderson (Houston, TX, USA) website [73]. Patient characteristics are reported in Table S3.

This study was reviewed and approved by the Institutional Review Board (IRB) of Samsung Medical Center, Seoul, Korea (IRB No: 2019-08-042), and was conducted in accordance with the Declaration of Helsinki. 

### 4.2. Isolation and Characterization of Extracellular Vesicles

Blood samples were collected prior to curative operation after completion of NAC. The samples were incubated to induce clotting at room temperature for 30 min and then centrifuged at 3000 rpm for 20 min. The collected serum was stored at −80 °C until use. EV purification was performed using the sequential ultracentrifugation protocol, as follows: 2000× *g* (10 min); 10,000× *g* (30 min); and 100,000× *g* (60 min; MLA-130 rotor). A flow chart of the EV isolation procedure based on differential ultracentrifugation is presented in Figure S5. The isolation method details have been described in previous studies on exosomal cytokines [33,74]. 

To characterize the purified EVs using transmission electron microscopy (TEM), EVs mixed with 2% paraformaldehyde were dropped onto a copper grid for 20 min. The EVs were fixed again with 2.5% glutaraldehyde for 5 min, washed 10 times with distilled water, and then negatively stained with 1% uranyl acetate for 1 min. Next, the copper grids were observed using a Hitachi 7700 transmission electron microscope operated at 80 kV. The diameter and particle number of the purified EVs were determined by NTA using a NanoSight model NS300 (Malvern Instruments, Malvern, UK). Data analysis was performed with the NTA v3.4 software. The following settings were used for data acquisition: camera level 16; acquisition time 30 s; and detection threshold 3. The isolated EVs were validated by detecting the presence of EV markers (CD63, CD9, and Tsg101) and the absence of negative markers (Calnexin, GRP94, and GAPDH) using western blot analysis.

### 4.3. Western Blot

Proteins were extracted using 10xRIPA buffer (Cell Signaling Technology, Danvers, MA, USA). Ten micrograms of total cell lysate or EVs was separated on 4–12% Bis-Tris gel (Invitrogen, Waltham, MA, USA) and transferred onto a PVDF membrane (Merck Millipore, Burlington, MA, USA). The membranes were probed using primary antibodies against the following molecules at 4 °C overnight: CD63 (SC5275, 1:1000) and CD9 (SC59140, 1:1000; both from Santa Cruz Biotechnology, Santa Cruz, CA, USA); Calnexin (2679, 1:1000), GRP94 (2104, 1:1000), and GAPDH (5174, 1:2000; all from Cell Signaling Technology); and Tsg101 (ab30871, 1:1000, Abcam, Cambridge, UK). The membranes were then incubated with an HRP-conjugated secondary antibody. All membranes were developed using an enhanced chemiluminescence system (Thermo Scientific, Waltham, MA, USA). 

### 4.4. Multiplex Immunoassay

The EV biomarker levels were measured with the ProcartaPlex Human Immune Monitoring 65-Plex Panel (Invitrogen) according to the manufacturer’s instructions. The analytes in the panel included 33 cytokines (G-CSF, GM-CSF, IFN-α, IFN-γ, IL-1α, IL-1β, IL-2, IL-3, IL-4, IL-5, IL-6, IL-7, IL-8, IL-9, IL-10, IL-12p70, IL-13, IL-15, IL-16, IL-17A, IL-18, IL-20, IL-21, IL-22, IL-23, IL-27, IL-31, LIF, M-CSF, MIF, TNF-α, TNF-β, and TSLP), 18 chemokines (BLC, ENA-78, Eotaxin, Eotaxin-2, Eotaxin-3, fractalkine, GROα, IP-10, I-TAC, MCP-1, MCP-2, MCP-3, MDC, MIG, MIP-1α, MIP-1β, MIP-3α, and SDF-1α), 6 growth factors/regulators (FGF-2, HGF, MMP-1, NGF-β, SCF, VEGF-A), and 8 soluble receptors (APRIL, BAFF, CD30, CD40L, IL-2R, TNF-R2, TRAIL, and TWEAK). Analyte measurement was performed using Bio-Plex200 (Bio-Rad Laboratories, Hercules, CA, USA), following the instruction manual. The analyte values were calculated from the standard curves using the 5-parameter logistic regression model. The full names of the analytes are presented in Table S1.

### 4.5. Statistical Analysis

Data were analyzed using SPSS software (version 25.0, IBM Corp., Armonk, NY, USA), GraphPad Prism 5 (GraphPad Software, La Jolla, CA, USA), and R v4.3.1. Overall survival (OS) was defined as the duration between curative surgery and death. Kaplan–Meier plots for the groups were compared with the log-rank test. Univariate and multivariate analyses for OS were performed with a Cox proportional hazards model to obtain the hazard ratio (HR) and 95% confidence interval (CI). Receiver operating characteristic (ROC) analysis was performed to calculate the AUCs, sensitivities, and specificities of cytokines levels for predicting overall survival. The optimal cutoff points for circulating cytokines were determined using the maximum value of the Youden index (sensitivity plus specificity minus 1). Categorical variables were presented as counts and proportions and were compared using the chi-square test. The difference between the two groups was evaluated using the Mann–Whitney *U* test. The Kruskal–Wallis test was applied to compare three or more groups. Analysis of correlation between variables was assessed using Spearman correlation. Two-tailed *p*-values < 0.05 were considered statistically significant in all analyses.

## 5. Conclusions

In this study, we investigated the roles of EV-associated cytokines as prognostic biomarkers for long-term outcomes in TNBC patients who underwent NAC. We found that high levels of EV_APRIL, EV_CXCL13, and EV_VEGF-A were correlated with poor overall survival. Since residual disease after NAC is associated with a poor prognosis, we further evaluated the role of selected biomarkers as prognostic values in patients with residual disease (non-pCR, RCB II/III, and stages II/III). In the non-pCR group, we also demonstrated that patients with high EV_APRIL, EV_CXCL13, and EV_VEGF-A levels had shorter overall survival than those with low levels. In all advanced subgroup analyses, an elevated EV_CXCL13 level was an independent predictor of poor OS. In addition, we measured the concentrations of soluble APRIL, CXCL13, and VEGF-A in the patients’ serums and compared them with those in EVs. The results showed a strong correlation between soluble VEGF-A and EV_VEGF-A. In addition, patients with high soluble VEGF-A levels also had poor survival. In contrast, we could not detect a significant correlation between the levels of soluble CXCL13 and EV_CXCL13. Our findings suggest that EV_APRIL, EV_CXCL13, and EV_VEGF-A may serve as biomarkers to predict overall survival in TNBC patients under neoadjuvant therapy. This information could be used to identify high-risk patients after NAC and to guide future treatment decisions. However, further prospective studies with large samples are warranted to validate the utility of these potential biomarkers in TNBC treated with NAC.

## Figures and Tables

**Table 1:** Patient characteristics and clinical outcomes.

Characteristics	Total (*n* = 190), *n* (%)	5Y OS (%)	*p*-Value
Age (years)			
<40	37 (19.5)	81.1	0.498
40–50	62 (32.7)	87.1	
>50	91 (47.9)	89.0	
Menopausal status			
Postmenopausal	87 (45.8)	89.7	0.584
Perimenopausal	1 (0.6)	100	
Premenopausal	100 (52.7)	85.0	
Unknown	2 (1.1)	0	
Clinical stage			
II	94 (49.5)	95.7	<0.001
III	96 (50.6)	78.1	
Clinical T stage			
1	17 (9)	88.2	<0.001
2	134 (70.6)	89.6	
3	37 (19.5)	81.1	
4	2 (1.1)	0	
Clinical N stage			
0	40 (21.1)	95.0	<0.001
1	64 (33.7)	95.3	
2	53 (27.9)	86.8	
3	33 (17.4)	60.6	
Postoperative pathologic stage			
N/A	65 (34.3)	96.9	<0.001
IA	56 (29.5)	96.4	
IB	5 (2.7)	100	
IIA	31 (16.4)	93.5	
IIB	13 (6.9)	53.8	
IIIA	13 (6.9)	38.5	
IIIC	7 (3.7)	28.6	
Nuclear grade (NG)			
N/A	57 (30)	94.7	0.022
2	17 (9)	70.6	
3	116 (61.1)	85.3	
Histologic grade (HG)			
N/A	57 (30)	94.7	<0.001
1	1 (0.6)	0	
2	31 (16.4)	90.3	
3	82 (43.2)	78.0	
Unknown	19 (10)		
RCB class			
0	65 (34.3)	96.9	<0.001
I	21 (11.1)	100	
II	71 (37.4)	90.1	
III	25 (13.2)	36.0	
Unknown	8 (4.3)		

Overall survival (OS) was determined via Kaplan–Meier analysis and the log-rank test. *p*-values ≤ 0.05 were considered significant. Abbreviations: RCB, residual cancer burden; NA, not accessed due to pathologic CR or insufficient residual tumor tissue.

**Table 2:** Receiver operating characteristic (ROC) analysis of the selected biomarkers for OS.

Parameters	Cutoff	Sensitivity	Specificity	Youden Index	AUC (95% CI)	*p*-Value
EV_APRIL	81.28	0.600	0.618	0.218	0.630 (0.505–0.756)	0.036
EV_CXCL13	50.33	0.760	0.539	0.299	0.671 (0.548–0.793)	0.006
EV_VEGFA	4.27	0.760	0.606	0.366	0.698 (0.584–0.813)	0.001

**Table 3:** Associations of EV_APRIL, EV_CXCL13, and EV_VEGF-A levels with clinical characteristics.

	EV_APRIL			EV_CXCL13			EV_VEGF-A		
Characteristics	Low *n* (%)	High *n* (%)	*p*	Low *n* (%)	High *n* (%)	*p*	Low *n* (%)	High *n* (%)	*p*
Age (years)									
<40	25 (22)	12 (15)	0.492	19 (20)	18 (19)	0.450	23 (22)	14 (17)	0.618
40–50	35 (31)	27 (35)		27 (28)	35 (37)		35 (33)	27 (32)	
>50	52 (46)	39 (50)		49 (52)	42 (44)		48 (45)	43 (51)	
Menopausal status									
postmenopause	50 (45)	37 (49)	0.630	48 (51)	39 (42)	0.279	47 (44)	40 (49)	0.584
perimenopause	1 (1)	0 (0)		1 (1)	0 (0)		1 (1)	0 (0)	
premenopause	61 (54)	39 (51)		46 (48)	54 (58)		58 (55)	42 (51)	
Clinical Stage									
II	62 (55)	32 (41)	0.052	52 (55)	42 (44)	0.147	61 (58)	33 (39)	0.012 *
III	50 (45)	46 (59)		43 (45)	53 (56)		45 (42)	51 (61)	
cStage T									
1	7 (6)	10 (13)	0.137	9 (9)	8 (8)	0.506	10 (9)	7 (8)	0.299
2	82 (73)	52 (67)		66 (69)	68 (72)		78 (74)	56 (67)	
3	23 (21)	14 (18)		20 (21)	17 (18)		18 (17)	19 (23)	
4	0 (0)	2 (3)		0 (0)	2 (2)		0 (0)	2 (2)	
cStage N									
1	25 (22)	15 (19)	0.351	26 (27)	14 (15)	0.180	23 (22)	17 (20)	0.004 *
2	42 (38)	22 (28)		31 (33)	33 (35)		44 (42)	20 (24)	
3	29 (26)	24 (31)		24 (25)	29 (31)		29 (27)	24 (29)	
4	16 (14)	17 (22)		14 (15)	19 (20)		10 (9)	23 (27)	
Pathologic Stage									
0	44 (39)	21 (27)	0.01 *	39 (41)	26 (27)	0.060	39 (37)	26 (31)	0.053
I	41 (37)	20 (26)		32 (34)	29 (31)		40 (38)	21 (25)	
II	18 (16)	26 (33)		18 (19)	26 (27)		19 (18)	25 (30)	
III	9 (8)	11 (14)		6 (6)	14 (15)		8 (8)	12 (14)	
RCB Class									
0	44 (41)	21 (28)	0.055	39 (43)	26 (29)	0.012 *	39 (39)	26 (31)	0.067
1	13 (12)	8 (11)		14 (15)	7 (8)		14 (14)	7 (8)	
2	41 (38)	30 (40)		31 (34)	40 (44)		38 (38)	33 (40)	
3	9 (8)	16 (21)		7 (8)	18 (20)		8 (8)	17 (20)	

A *p*-value < 0.05 is significant. * denote significance. Abbreviations: RCB, residual cancer burden.

**Table 4:** Risk factor analysis for overall survival (OS) in patients with non-pCR, RCB II/III, and stages II/III.

	Non-pCR (*n* = 125)				RCB II/III (*n* = 96)				Stages II/III (*n* = 64)			
	Univariate Analysis		Multivariate Analysis		Univariate Analysis		Multivariate Analysis		Univariate Analysis		Multivariate Analysis	
Variable	HR (95% CI)	*p*	HR (95% CI)	*p*	HR (95% CI)	*p*	HR (95% CI)	*p*	HR (95% CI)	*p*	HR (95% CI)	*p*
Age (years)		0.172		**0.033**		0.173		**0.041**		**0.001**		**0.027**
<40	2.63 (0.95–7.26)	0.062	4.76 (1.51–14.94)	**0.008**	2.58 (0.94–7.13)	0.067	4.57 (1.45–14.45)	**0.010**	10.99 (3.46–34.95)	**<0.001**	5.59 (1.64–19.11)	**0.006**
40–50	1.45 (0.55–3.88)	0.453	1.6 (0.58–4.41)	0.36	1.31 (0.49–3.48)	0.592	1.56 (0.57–4.3)	0.387	1.66 (0.6–4.57)	0.329	1.88 (0.66–5.4)	0.238
≥50	ref		ref		ref		ref		ref		ref	
Stage (AJCC 7th)		**<0.001**		**<0.001**		**<0.001**		**<0.001**		**<0.001**		**0.001**
1A	ref		ref		ref		ref					
1B	0 (0–0)	0.987	0 (0–0)	0.982	0 (0–0)	0.987	0 (0–0)	0.987				
2A	1.81 (0.25–12.85)	0.553	2.81 (0.36–21.89)	0.323	1.17 (0.17–8.33)	0.873	2.43 (0.31–19.22)	0.399	ref		1.00	
2B	16.9 (3.41–83.83)	**0.001**	33.05 (5.99–182.32)	**<0.001**	9.84 (1.98–48.8)	**0.005**	24.4 (4.3–138.48)	**<0.001**	9.3 (1.88–46.15)	0.006	11.61 (2.28–59.15)	**0.003**
3A	24.47 (5.16–116)	**< 0.001**	27.78 (5.57–138.63)	**<0.001**	14.25 (3.01–67.54)	**0.001**	20.89 (4.16–104.9)	**<0.001**	13.44 (2.83–63.78)	0.001	9.55 (1.92–47.57)	**0.006**
3C	37.38 (7.17–194.83)	**< 0.001**	46.76 (8.45–258.64)	**<0.001**	21.77 (4.18–113.43)	**<0.001**	34.86 (6.21–195.63)	**<0.001**	20.7 (3.97–107.98)	<0.001	17.15 (2.92–100.77)	**0.002**
EV_APRIL		**0.039**		-		0.056		-		0.302		-
Low	ref		-		ref		-		ref		-	
High	2.47 (1.05–5.83)		-		2.31 (0.98–5.46)		-		1.61 (0.65–4)		-	
EV_CXCL13		**0.008**		**0.011**		**0.023**		**0.014**		**0.048**		**0.019**
Low	ref		ref		ref		ref		ref		1.00	
High	4.26 (1.45–12.54)		4.33 (1.39–13.47)		3.5 (1.19–10.31)		4.17 (1.34–13.01)		3.00 (1.01–8.93)		4.05 (1.25–13.06)	
EV_VEGF-A		**0.007**		-		**0.026**		-		0.069		-
Low	ref		-		ref		-		ref		-	
High	3.6 (1.42–9.13)		-		2.87 (1.13–7.28)		-		2.54 (0.93–6.95)		-	

Abbreviations: HR, hazard ratio; CI, confidence interval; RCB, residual cancer burden. Significant *p*-values (<0.05) are bolded.

## Data Availability

Data are contained within the article or supplementary material. Additional information is available on request from the corresponding author.

## Supplementary Materials

The following supporting information can be downloaded at https://www.mdpi.com/article/10.3390/ijms242115576/s1.
