# Supplementary material for: A Retrospective Exploratory Analysis for Serum Extracellular Vesicles Reveals APRIL (TNFSF13), CXCL13, and VEGF-A as Prognostic Biomarkers for Neoadjuvant Chemotherapy in Triple-Negative Breast Cancer
Source: Int J Mol Sci. 2023 Oct 25;24(21):15576. doi: 10.3390/ijms242115576 (PMC10647725; doi:10.3390/ijms242115576)
Supplement: Supplementary file 1 [file ijms-24-15576-s001.zip › 0 2_2 Supplementary Materials.pdf]

Supplementary Materials

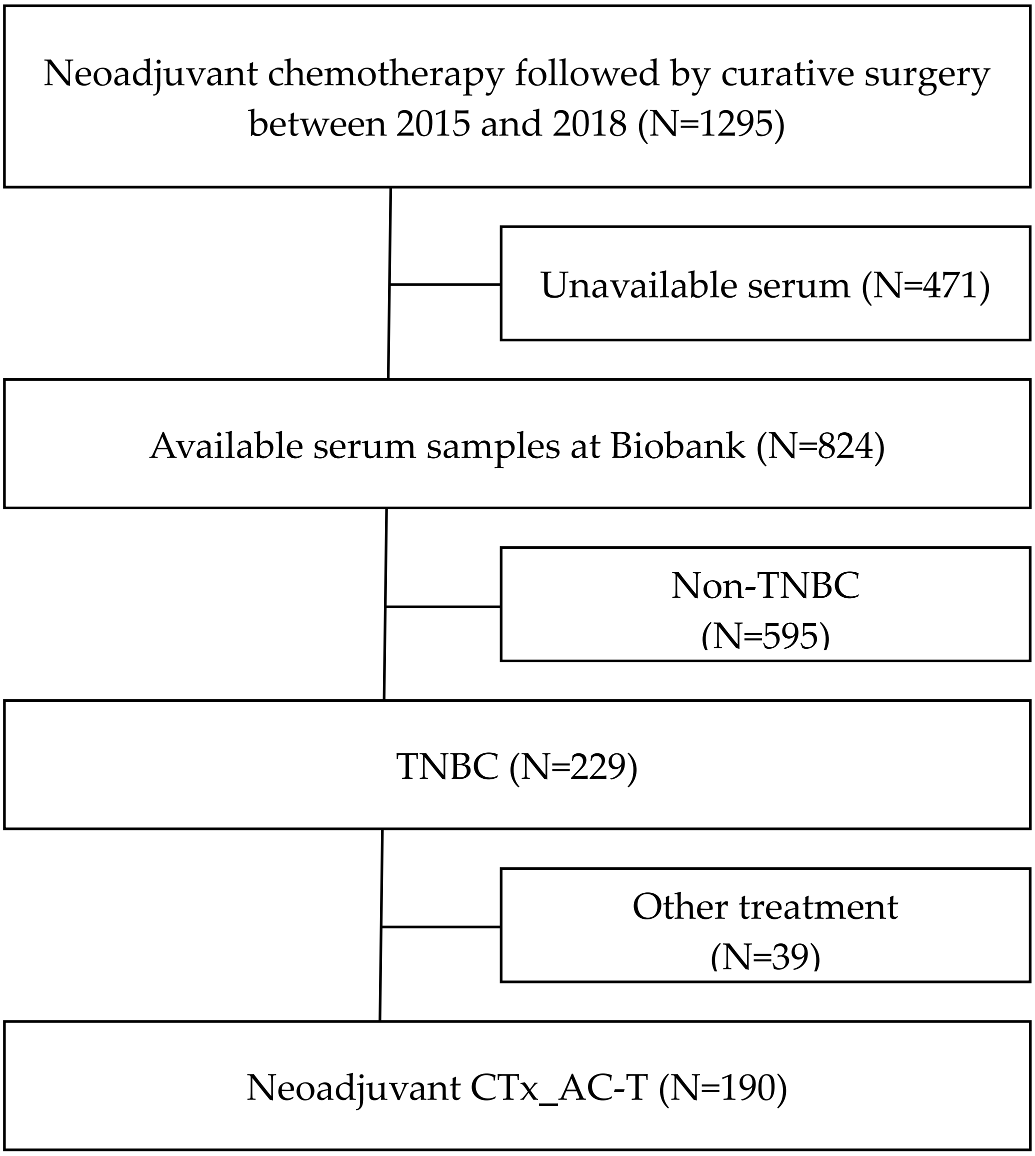

Figure S1. Consort diagram

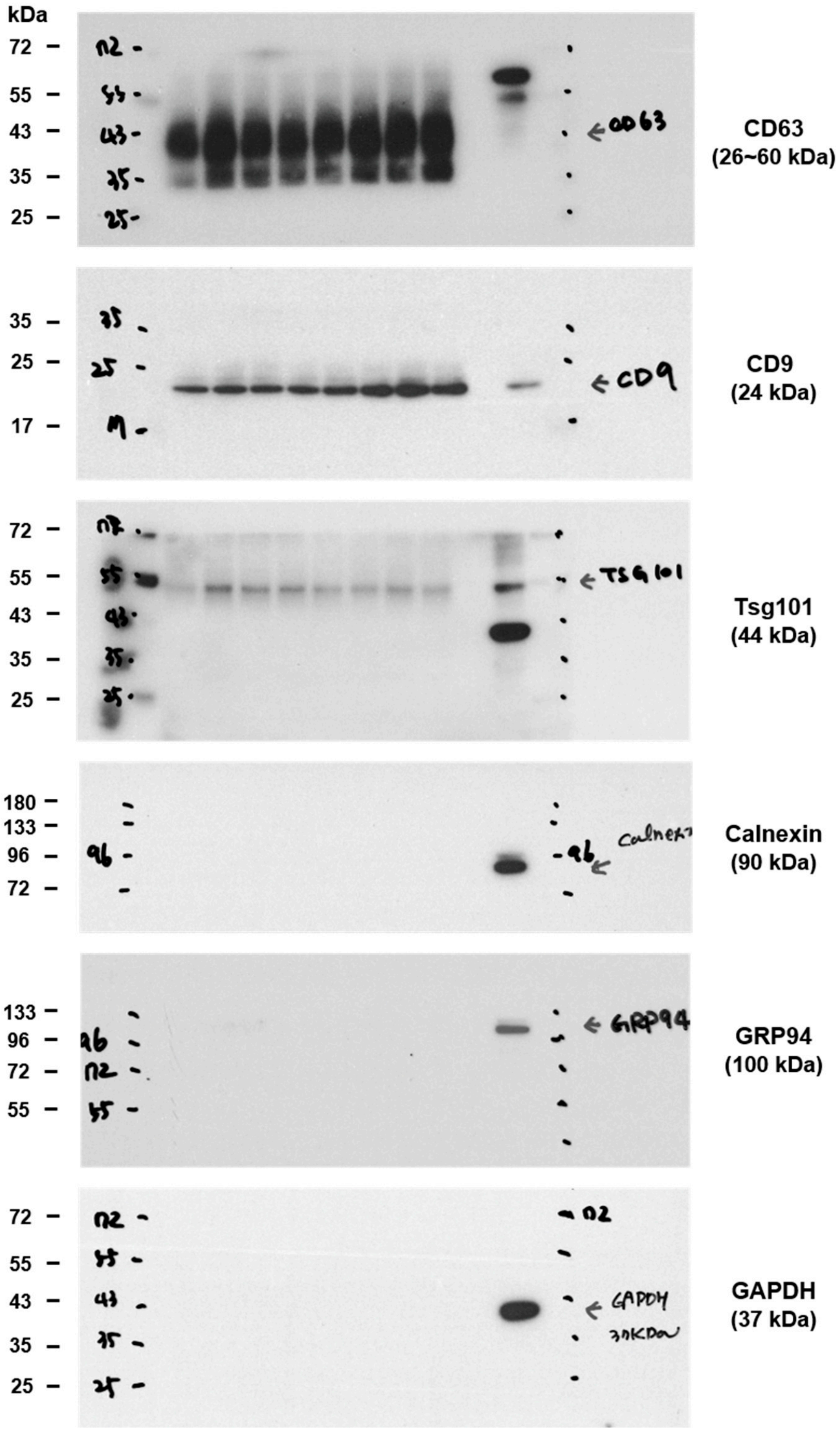

Figure S2. Uncut blots for Figure 1C

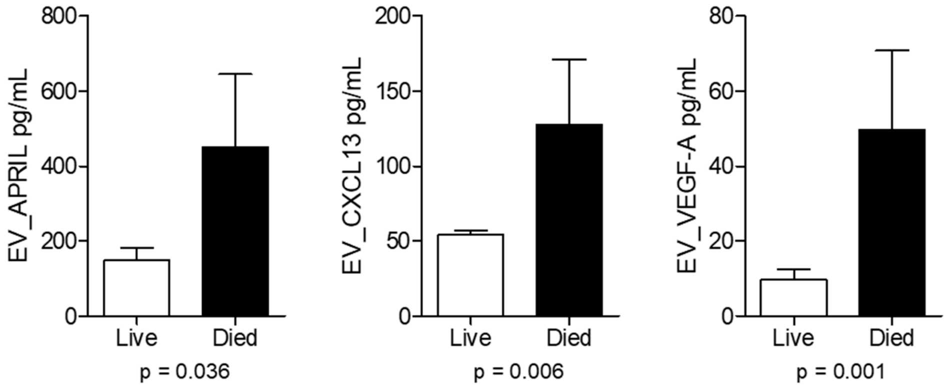

Figure S3. Biomarker expression levels according to survival. Mann-Whitney U-test was used to compare differences between the two groups.

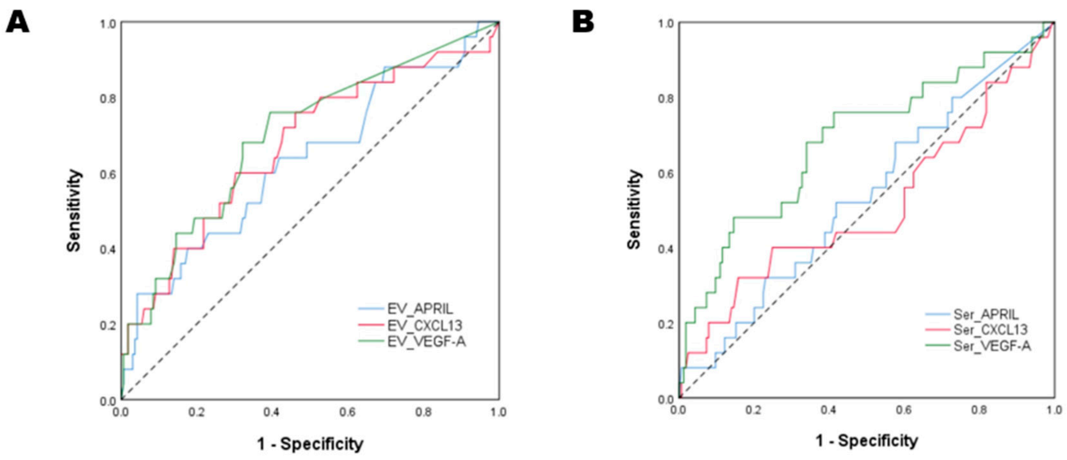

Parameters	Cutoff	Sensitivity	Specificity	Youden index	AUC (95% CI)	*p*-value
Ser_APRIL	550.50	0.680	0.424	0.104	0.537 (0.416-0.659)	0.549
Ser_CXCL13	74.50	0.320	0.842	0.162	0.511 (0.374-0.648)	0.861
Ser_VEGFA	381.50	0.760	0.588	0.348	0.684 (0.561-0.807)	0.003

Figure S4. Receiver operator characteristic analysis of cytokine levels in EVs (A) and serum (B) for overall survival. Abbreviations: AUC, area under the curve; CI, confidence interval.

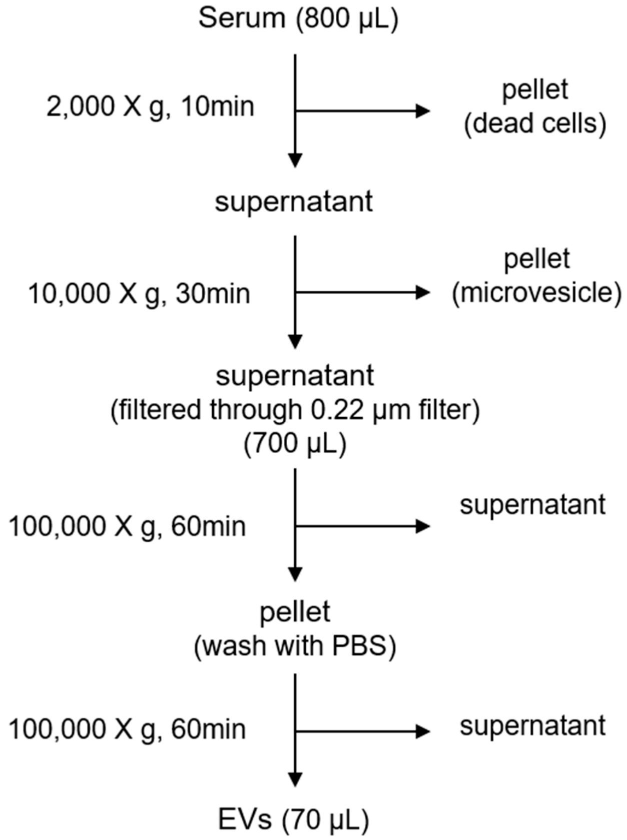

Figure S5. Flow chart of the EV isolation procedure based on differential ultracentrifugation.

Table S1. Full name for each target in the ProcartaPlex Immune Monitoring 65-Plex Panel

Table S2. Correlation analysis among biomarker concentrations in EVs and serum

Table S3. Clinicopathological characteristics of 190 TNBC patients
